# Significance of co-positivity for anti-dsDNA, -nucleosome, and -histone antibodies in patients with lupus nephritis

**DOI:** 10.1080/07853890.2023.2187076

**Published:** 2023-03-10

**Authors:** Sung-Eun Choi, Dong-Jin Park, Ji-Hyoun Kang, Shin-Seok Lee

**Affiliations:** Division of Rheumatology, Department of Internal Medicine, Chonnam National University Medical School & Hospital, Gwangju, Republic of Korea

**Keywords:** Lupus nephritis, autoantibodies, pathology

## Abstract

**Objective:**

The aim of this study was to define the clinical, histopathologic, and prognostic features associated with simultaneous positivity for anti-dsDNA, -nucleosome, and -histone antibodies (3-pos) in Korean patients with biopsy-proven lupus nephritis (LN).

**Methods:**

The 102 patients included in the study had undergone kidney biopsy prior to the start of induction treatment, were treated with immunosuppressives, and followed-up for >12 months.

**Results:**

In total, 44 (43.1%) of the 102 LN patients were 3-pos. Patients with 3-pos had a higher SLEDAI-2K score (*p* = .002), lower lymphocyte count (*p* = .004), and higher rates of proteinuria > 3.5 g/24 h (*p* = .039) and positivity for urinary sediments (*p* = .005) at the time of renal biopsy than non-3-pos patients. 3-pos patients had a more proliferative form of LN (*p* = .045) in the renal histopathologic findings, and as co-positivity gradually increased from 0 to 3, the total activity score in the renal biopsy findings increased significantly (*p* = .033). In addition, 3-pos patients had a more rapid eGFR decline than non-3-pos patients after a follow-up of 83.2 months (*p* = .016).

**Conclusions:**

Our findings suggest that 3-pos is related to severe LN and that 3-pos patients are more likely to experience a rapid decline of renal function than non-3-pos patients.

**KEY MESSAGE**
- Patients with co-positivity for anti-dsDNA, -nucleosome, and -histone antibodies (3-pos) had higher disease activity and a worse renal histopathology than those without co-positivity.
- 3-pos patients had a more rapid decline of renal function than non-3-pos patients.

## Introduction

Systemic lupus erythematosus (SLE) is a chronic autoimmune disorder characterized by the presence of autoantibodies and immune-complex-mediated tissue damage involving the kidney, skin, joints, bone marrow, nervous system, and other organs. Lupus nephritis (LN), i.e. the renal involvement of SLE, occurs in up to 60% of patients with SLE [1]. Although advanced treatment using new immunosuppressive agents, including mycophenolate mofetil (MMF), calcineurin inhibitors, and rituximab, has improved the outcomes of LN patients, the risk of developing the end-stage renal disease at 5, 10, and 15 years is still high (11%, 17% and 22%, respectively) [2]. This permanent renal damage in LN is significantly related to increases in morbidity and mortality, and to a higher risk of cardiovascular disease, which is the leading cause of death in these patients [3]. Therefore, measures to predict the progression to renal dysfunction in LN patients are needed, as they will permit more accurate assessments of SLE disease activity and aid treatment.

Autoantibodies in SLE have diagnostic relevance and are associated with certain clinical manifestations and disease activity [4]. In particular, several autoantibodies play a role in the development and progression of LN. Pathological analyses of renal tissues have identified autoantibodies targeted against antigens such as DNA, histone and nucleosome [5,6]. The existence of anti-dsDNA, anti-nucleosome and/or anti-histone antibodies is associated with proliferative glomerulonephritis and LN activity [7–9]. An association of the presence of a single autoantibody type with the development of LN has been demonstrated in LN patients, and the presence of multiple autoantibodies has been suggested in the development and progression of LN. The coexistence of anti-dsDNA, anti-nucleosome and anti-histone antibodies is frequently observed in LN patients, and simultaneous positivity for these autoantibodies is presumed to have a greater risk of developing proliferative form of LN and worse renal outcome compared to single positivity. Thus, we explored whether LN patients with co-positivity with anti-dsDNA, anti-nucleosome, and anti-histone (3-pos) antibodies have higher disease activity and more active renal histopathology than those without co-positivity. We also evaluated whether the higher global and renal activity is associated with a worse renal outcome.

## Patients and methods

### Study design and population

The KORean lupus NETwork (KORNET) is a tertiary care center-based registry that investigates the outcomes of Korean patients with SLE. Between January 2005 and January 2017, 420 patients with SLE were enrolled in the cohort. All patients fulfilled the 1997 revised criteria for the classification of SLE [10]. As a subcohort of the KONET registry, we established an inception cohort of LN patients to evaluate their renal outcomes and long-term prognosis. The inception cohort enrolled patients at the time of renal biopsy, regardless of prior diagnosis of SLE. Analyses were limited to patients who had visited Chonnam National University Hospital within 6 months of LN diagnosis. Patients were included if they had confirmed LN based on renal biopsy findings and at least 1-year of the follow-up data. Among the 250 patients followed up at Chonnam National University Hospital, 164 were diagnosed with LN. Among these patients, 102 were included after excluding 32 patients with less than 1 year of follow-up, 15 with advanced comorbidities or other diseases associated with kidney dysfunction, such as diabetic kidney disease or primary kidney disease, 8 with inadequate medical records and 7 aged under 18 years. All patients were followed up at 1- to 3-month-intervals, from the time of renal biopsy until at least 1 year later, *via* the KORNET database. The patients were followed up using the Internet-based Clinical Research and Trial management system (iCReaT; http://icreat.nih.go.kr), which is a data management system established by the Centers for Disease Control and Prevention, Ministry of Health and Welfare, Republic of Korea (iCReaT Study No. C140018). The patients were classified according to whether they or not they exhibited simultaneous positivity for anti-dsDNA, -nucleosome, and -histone antibodies (3-pos and non-3-pos groups, respectively). This study was conducted in accordance with the Declaration of Helsinki, and was approved by the Institutional Review Board of Chonnam National University Hospital (approval no. CNUN-2014-239). Informed consent was obtained at the time of registry enrollment.

### Patient data collection

The characteristics of the patients were evaluated at baseline, i.e. at the time of renal biopsy. Demographic data, including age at LN onset, sex, disease duration at LN onset, and comorbidities, were collected. The medication history of hypertension and diabetes mellitus were also obtained. The SLE Disease Activity Index-2000 (SLEDAI-2K) was determined at the time of the biopsy [11].

Laboratory data were obtained, including the white blood cell count, lymphocyte count, hemoglobin level, platelet level, erythrocyte sedimentation rate (ESR), C-reactive protein (CRP), serum albumin level, serum creatinine level, urinalysis results and urinary protein excretion (g/day), at the time of renal biopsy. The presence of urinary sediments was defined as >5 RBCs and >5 WBCs per high power field and/or cellular casts, as recommended in the ACR 2006 clinical trial criteria [12]. The estimated glomerular filtration rate (eGFR) was calculated according to the equation used in the Modification of Diet in Renal Disease (MDRD) study: eGFR (mL/min/1.73 m^2^) = 186 × (S_Cr_ [mg/dL])^−1154^ × (age)^−0.203^ × (0.742 if female). The anti-nuclear antibody (ANA) test was performed on a Helios automated indirect immunofluorescence assay analyzer using the ANA Hep-2 standard kit (Aesku Diagnostics, Wendelsheim, Germany). The sensitivity and specificity of this assay were reported as 73.3% and 99.4%, respectively [13]. Autoantibodies including Smith, ribonucleoprotein, Ro/SS-A, La/SS-B, nucleosome, histone, and ribosomal-P autoantibodies were assessed by standalone ELISA using a commercial kit (Alegria; ORGENTEC Diagnostika GmbH, Mainz, Germany). Anti-dsDNA antibody was determined by radioimmunoassay using a commercial kit (Trinity Biotech PLC; Wicklow, Ireland). Antiphospholipid antibody (aPL), including lupus anticoagulant (LAC), anti-cardiolipin (aCL), and anti-beta_2_-glycoprotein I (β_2_GPI) was also measured. LAC was evaluated using the modified Russell’s viper venom time test, with confirmation by mixing test and IgG/M aCL and IgG/M anti- β_2_GPI antibodies were analyzed by ELISA (Alegria; ORGENTEC Diagnostika, Mainz, Germany), and the results were considered positive if the titer was medium to high. In this study, patients were considered aPL-positive if they were positive for at least one of these autoantibodies.

The results of the renal biopsy were re-classified by two blinded renal pathologists based on the 2004 ISN/RPS criteria [14]. Activity and chronicity indices were evaluated based on the scoring systems of the US National Institutes of Health [15]. Patients with mixed-type LN were considered to have the predominant type. For example, type III + IV cases were classified as type III, and type IV + V cases as type IV.

### Treatment and definition of remission

Treatment was at the discretion of the responsible rheumatologist. As an induction treatment, corticosteroids with high-dose intravenous cyclophosphamide (CYC; 500–1000 mg/m^2^ body surface area every month, 6 times) or corticosteroids with an oral immunosuppressant such as mycophenolate mofetil (MMF; up to 3 g/d), azathioprine (AZA), cyclosporine (CsA), or tacrolimus (TAC) was used. For maintenance therapy, quarterly intravenous CYC or oral immunosuppressant was prescribed. Prednisolone of 30–60 mg/d was given with or without intravenous methylprednisolone pulse therapy (500–1000 mg/d × 3 d). Patients with LN types I and II were treated with low-to-moderate doses of prednisone (0.5 mg/kg/d) alone and oral immunosuppressants were added when the renal response was unsatisfactory. In general, induction treatment was administered for ∼6 months, based on the treating rheumatologist’s clinical decision.

Treatment responses after 12 months were defined using the ACR 2006 clinical trial criteria [12]. A complete renal response (CR) was defined as an eGFR >90 mL/min/1.73 m^2^, a urine protein-to-creatinine ratio <0.2, and inactive urinary sediment. A partial response (PR) was defined as a stable eGFR, the urinary protein-to-creatinine ratio in the range of 0.2–2.0, and no urinary sediment. No response (NR) was defined as failure to meet the CR or PR. Renal relapse was defined based on clinical manifestations indicating *a* > 25% decline in the eGFR, ≥50% increase in proteinuria, or the appearance of urinary sediment in a patient who had previously experienced a CR or PR. CKD was defined as an eGFR <60 mL/min/1.73 m^2^ for ≥3 months.

The drugs used as induction and maintenance therapies were recorded, as were all medications taken for >3 months prior to LN onset, such as hydroxychloroquine and prednisolone (>5 mg/d). Continuous use of hydroxychloroquine (for >8 months during the 1-year follow-up period after LN onset), and the use of angiotensin-converting enzyme inhibitors or angiotensin receptor blockers, were also documented.

### Statistical analysis

Statistical analyses were performed using SPSS software (ver. 18.0; SPSS Inc., Chicago, IL, USA). The results are expressed as the mean ± standard deviation for continuous variables and as a percentage for categorical variables. Baseline characteristics, laboratory findings, autoantibodies and complements levels, and treatment and clinical outcomes were compared between the 3-pos and non-3-pos groups using the Mann–Whitney *U* test for continuous variables and the chi-square test or Fisher’s exact test for categorical variables. The association of each autoantibody with renal biopsy findings was evaluated using the Mann–Whitney *U* test, while the association of co-positivity with renal biopsy findings was analyzed using a one-way analysis of variance (ANOVA) with the Bonferroni post hoc test. The chi-square test or Fisher’s exact test was used to analyze the positivity of each autoantibody according to the ISN/RPS classification. A *p*-value <.05 was considered to indicate statistical significance.

## Results

A total of 102 LN patients were finally included in this study. The mean age at LN onset was 35.4 ± 10.9 years and 89 patients (87.3%) were female. The mean disease duration at LN onset was 32.8 ± 51.7 months. At the time of LN onset, 39 patients (38.2%) had hypertension and 4 (3.9%) had diabetes mellitus. The mean SLEDAI-2K score was 12.5 ± 4.8. Among the 102 patients with biopsy-proven LN patients, 44 (43.1%) showed positivity for anti-dsDNA, -nucleosome, and -histone (3-pos). For the non-renal lupus patients, after excluding 14 patients with missing autoantibody data, 11 of 72 patients (15.3%) were 3-pos. Thus, LN patients had a significantly higher proportion of 3-pos than non-renal patients (*p* < .001).

The baseline characteristics and laboratory findings of the 3-pos and non-3-pos LN patients are shown in Table 1. The average age and disease duration at LN onset did not significantly differ between the two groups. With respect to the laboratory findings, the lymphocyte count was significantly lower in the 3-pos than non-3-pos group (*p* = .004). There was no difference in the 24-h urine protein level between the two groups, but proteinuria >3.5 g/24 h and urine sediments were significantly more common in the 3-pos group (*p* = .039 and *p* = .005, respectively). Other laboratory findings, including ESR, CRP, serum creatinine, and eGFR, did not significantly differ between the two groups. The mean SLEDAI-2K score (i.e. disease activity) at the time of renal biopsy was significantly higher in the 3-pos group than in the non-3-pos group (14.2 ± 4.67 vs. 11.3 ± 4.87, respectively; *p* = .002). The renal SLEDAI-2K score was significantly higher in the 3-pos group than in the non-3-pos group (*p* = .002). However, there was no significant difference in the non-renal SLEDAI-2K score between the groups (*p* = .117).

**Table 1.** Baseline characteristics and laboratory findings of lupus nephritis (LN) patients according to their 3-pos status.

	All patients (*n* = 102)	3-pos (*n* = 44)	Non 3-pos (*n* = 58)	*p* value
Age at LN onset, years	35.4 ± 10.9	32.9 ± 9.87	37.2 ± 11.3	.058
Female, *n* (%)	89 (87.3 %)	39 (88.6)	50 (86.2)	.479
Disease duration at LN onset, months	32.8 ± 51.7	36.1 ± 50.9	30.2 + 52.7	.385
Hypertension at LN onset, *n* (%)	39 (38.2 %)	14 (31.8)	25 (43.9)	.218
Diabetes mellitus at LN onset, *n* (%)	4 (3.9%)	1 (2.3)	3 (5.3)	.412
Laboratory findings				
White blood cells, /mm^3^	6340.7 ± 3856.9	5511.4 ± 3029.7	6969.9 ± 4265.7	.085
Lymphocytes, /mm^3^	1279.8 ± 1088.1	955.4 ± 459.6	1525.9 ± 1339.9	.004
Hemoglobin, g/dL	10.4 ± 2.07	10.1 ± 1.94	10.6 ± 2.15	.232
Platelets, 10^3^/ul	209.4 ± 103.6	205.4 ± 99.9	211.8 ± 108.5	.834
ESR, mm/hr	47.1 ± 34.8	42.1 ± 32.6	46.7 ± 36.6	.751
CRP, mg/dL	0.68 ± 0.92	0.60 ± 0.60	0.74 ± 1.10	.764
Albumin, mg/dL	3.15 ± 0.92	3.17 ± 0.92	3.12 ± 0.92	.695
Serum Cr, mg/mL	0.95 ± 0.64	0.84 ± 0.42	0.98 ± 0.62	.217
eGFR, ml/min/1.72m^2^	115.1 ± 67.7	115.9 ± 50.9	111.2 ± 74.9	.191
Proteinuria, g/24 h	3.55 ± 2.87	3.78 ± 3.04	3.38 ± 2.81	.466
Proteinuria > 3.5 g/24 h	35 (34.3%)	20 (45.5%)	15 (25.9)	.039
Urine sediments	63 (61.8%)	34 (77.3)	29 (50.0)	.005
SLEDAI-2K score	12.5 ± 4.8	14.2 ± 4.67	11.3 ± 4.87	.002
Renal SLEDAI-2K score	6.27 ± 2.07	7.00 ± 1.75	5.72 ± 2.13	.002
Non-renal SLEDAI-2K score	6.25 ± 4.33	7.18 ± 4.62	5.53 ± 4.00	.117

Unless otherwise indicated, the values are mean ± standard deviation. The presence of urinary sediments was defined as >5 RBCs and >5 WBCs per high power field and/or cellular casts. LN: lupus nephritis; 3-pos: simultaneous positivity of anti-dsDNA, -nucleosome and -histone antibodies; ESR: erythrocyte sedimentation rate; CRP: C-reactive protein; Cr: creatinine; eGFR: estimated glomerular filtration rate; SLEDAI-2K: Systemic Lupus Erythematosus Disease Activity Index-2000.

The autoantibody and complement levels in LN patients in the 3-pos and non-3-pos groups are shown in Table 2. As expected, a significantly larger proportion of patients were positive for anti-dsDNA, anti-nucleosome, and anti-histone in the 3-pos than non-3-pos group (all *p* < .001). In addition, significantly more patients were positive for anti-RNP in the 3-pos group (*p* = .019). By contrast, C3 levels were significantly lower in the 3-pos than non-3-pos group (*p* = .023).

**Table 2.** Autoantibodies and complement levels in LN patients according to 3-pos status.

	All patients (*n* = 102)	3-pos (*n* = 44)	Non 3-pos (*n* = 58)	*p* value
Autoantibodies				
Antinuclear, *n* (%)	99 (97.1%)	41 (95.3)	56 (98.2)	.401
Anti-dsDNA > 7, *n* (%)	86 (84.3%)	44 (100)	42 (74.2)	<.001
Anti-dsDNA	466.9 ± 1044.8	908.3 ± 1430.5	143.9 ± 332.4	.001
Anti-Sm, *n* (%)	45 (44.1%)	22 (50.0)	23 (39.7)	.297
Anti-RNP, *n* (%)	49 (48.0%)	27 (61.4)	22 (37.9)	.019
Anti-Ro/SS-A, *n* (%)	70 (68.6%)	26 (59.1)	44 (75.9)	.071
Anti-La/SS-B, *n* (%)	29 (28.4%)	11 (25)	18 (31)	.503
Anti-nucleosome, *n* (%)	69 (67.6%)	44 (100)	25(43.1)	<.001
Anti-histone, *n* (%)	54 (52.9%)	44 (100)	10 (17.2)	<.001
Anti-ribosomal P, *n* (%)	35 (34.3)	19 (43.2)	16 (27.6)	.100
Lupus anticoagulant, *n* (%)	16 (14.7%)	4 (9.5)	6 (11.1)	.538
Anti-phospholipid, *n* (%)	25 (26.9)	10 (25.0)	15 (28.3)	.722
Complement levels				
C3	57.1 ± 28.3	48.6 ± 23.9	63.2 ± 29.8	.023
C4	10.4 ± 8.27	9.73 ± 8.72	11.0 ± 7.95	.189

Unless otherwise indicated, the values are mean ± standard deviation. LN: lupus nephritis; 3-pos: simultaneous positivity for anti-dsDNA, -nucleosome, and -histone antibodies.

Table 3 and the supplementary table show the associations of anti-dsDNA, anti-nucleosome, and anti-histone antibodies with the renal biopsy findings. Endo-capillary hypercellularity and fibrinoid necrosis/karyorrhexis were higher in patients with than without anti-dsDNA positivity according to the activity index (*p* = .048 and *p* = .035, respectively). The rate of glomerular sclerosis according to the chronicity index was lower in patients with than without anti-dsDNA positivity (*p* = .016). Similarly, endo-capillary hypercellularity and fibrinoid necrosis/karyorrhexis were increased in patients with anti-nucleosome positivity compared to those without, according to the activity index as well as the total activity score (*p* = .016, *p* = .011 and *p* = .042, respectively). Endo-capillary hypercellularity, leukocyte infiltration, sub-endothelial hyaline deposits, and fibrinoid necrosis/karyorrhexis were increased in patients with anti-histone positivity compared to those without, according to the activity index as well as the total activity score (*p* = .016, *p* = .008, *p* = .009, *p* < .001, and *p* = .002, respectively). Thus, overall, as co-positivity gradually increased from 0 to 3, both the degree of activity, including endo-capillary hypercellularity, sub-endothelial hyaline deposits, fibrinoid necrosis/karyorrhexis, and cellular crescents, and the total activity score increased significantly (*p* = .016, *p* = .045, *p* = .002, *p* = .022, and *p* = .033, respectively). For the ISN/RNS classification, the proportion of patients with proliferative LN (types III and IV) increased significantly as the level of co-positivity increased (*p* = .045).

**Table 3.** Associations of autoantibodies with renal biopsy findings in patients with lupus nephritis.

	Anti-dsDNA			Anti-nucleosome			Anti-histone			Co-positivity (n)					
	Positive (*n* = 86)	Negative (*n* = 16)	*p* value	Positive (*n* = 69)	Negative (*n* = 33)	*p* value	Positive (*n* = 54)	Negative (*n* = 48)	*p* value	0 *n* = 8	1 *n* = 23	2 *n* = 27	3 *n* = 44	*p* for trend	*p* for trend*
Activity score	5.99 ± 4.39	3.69 ± 3.40	.078	6.25 ± 4.26	4.33 ± 4.21	.042	6.85 ± 4.20	4.25 ± 4.01	.002	1.88 ± 2.53	4.17 ± 3.81	6.26 ± 4.41	6.68 ± 4.29	.011	.033
Chronicity score	1.76 ± 1.39	2.50 ± 1.89	.125	1.84 ± 1.37	1.94 ± 1.75	.937	1.94 ± 1.37	1.79 ± 1.63	.447	2.75 ± 2.05	1.43 ± 1.53	2.15 ± 1.56	1.77 ± 1.25	.175	.525
Activity score (>12), *n* (%)	5 (31.3)	46 (53.5)	.086	38 (55.1)	13 (39.4)	.138	34 (66.7)	17 (35.4)	.005	1 (12.5)	8 (34.8)	16 (59.3)	26 (59.1)	.029	
Chronicity score (>4), *n* (%)	3 (18.8)	10 (11.6)	.331	8(11.6)	5 (15.2)	.614	6 (11.1)	7 (14.6)	.600	1 (12.5)	3 (13.0)	6 (22.2)	3 (6.8)	.311	
ISN/RPS classification, *n* (%)														.015	.045
I	8 (9.3)	3 (18.8)	.372	6 (8.7)	5 (15.2)	.328	2 (3.7)	9 (18.8)	.023	2 (25.0)	4(17.9)	3 (11.1)	2 (4.5)		
II	7 (8.1)	3 (18.8)	.189	4 (5.8)	6 (18.2)	.073	4 (7.4)	6 (12.5)	.510	3 (37.5)	3 (13.0)	0 (0.0)	4 (9.1)		
III	21 (24.4)	3 (18.8)	.757	19 (27.5)	5 (15.2)	.216	17 (31.5)	7 (14.6)	.061	0 (0.0)	3 (13.0)	9 (33.1)	12 (27.3)		
IV	43 (50.0)	7 (25.0)	.100	35 (50.7)	12 (36.4)	.206	29 (53.7)	18 (37.5)	.115	1 (12.5)	9(39.1)	13(48.1)	24(54.5)		
V	7 (8.1)	3 (18.8)	.189	5 (7.2)	5 (15.2)	.286	2 (3.7)	8 (16.7)	.043	2 (25.0)	4(17.9)	2 (7.4)	2(4.5)		

Unless otherwise indicated, the values are mean ± standard deviation.

* Bonferroni corrected *p*-value.

The treatment and clinical outcomes of LN patients according to the presence of 3-pos are shown in Table 4. The rate of use of medication before LN diagnosis and induction therapy after LN diagnosis did not differ between the 3-pos and non-3-pos groups, and nor did the clinical outcomes, including the 1-year CR, relapse, end-stage renal disease, and death. However, a rapid eGFR decline, defined as an absolute annual decline of eGFR ≥5 ml/min/1.73 m^2^ and eGFR at the last follow-up of <90 ml/min/1.73 m^2^ were more frequently seen in the 3-pos than non-3-pos group after a mean follow-up of 83.2 months (*p* = .016).

**Table 4.** Treatment and clinical outcomes of LN patients according to 3-pos status.

	All patients (*n* = 102)	3-pos (*n* = 44)	Non 3-pos (*n* = 58)	*p* value
Treatment before LN diagnosis				
Hydroxychloroquine	59 (57.8)	29 (65.9)	30 (50.8)	.151
Any immunosuppressive agent	20 (19.6)	12 (27.3)	8 (13.8)	.089
Treatment after LN diagnosis				
Hydroxychloroquine (>80% use during follow up)	77 (75.2)	35 (79.5)	42 (72.4)	.407
Induction therapy				.222
CYC	35 (34.3)	18 (40.9)	17 (29.3)	
Non-CYC	67 (65.7)	26 (59.1)	41 (70.7)	
Statin	35 (34.3)	13 (29.5)	22 (37.9)	.377
ACEi or ARB	83 (81.4)	37 (84.1)	46 (79.3)	.539
Clinical outcomes				
1-year CR	50 (49.0)	19 (43.2)	31 (53.4)	.304
Relapse	52 (51.0)	24 (54.5)	28 (48.3)	.530
Rapid eGFR progression*	51 (50.0)	28 (63.6)	23 (39.7)	.016
ESRD	11 (10.8)	6 (13.6)	5 (8.6)	.419
Death	3 (3.0)	1 (2.3)	2 (3.6)	.720

All values are reported as percentages. LN: lupus nephritis; 3-pos: simultaneous positivity of anti-dsDNA, -nucleosome and -histone antibodies; CYC: cyclophosphamide; ACEi: angiotensin-converting enzyme inhibitor; ARB: angiotensin receptor blocker; CR: complete renal response; eGFR: estimated glomerular filtration rate; ESRD: end-stage renal disease.

* Rapid eGFR progression is defined as an absolute annual decline of eGFR ≥5 ml/min/1.73 m^2^ each year and an eGFR at the last follow-up of <90 ml/min/1.73 m^2^.

## Discussion

This study examined the clinical, histopathologic, and prognostic significance of simultaneous positivity for anti-dsDNA, -nucleosome, and -histone antibodies (3-pos) in patients with biopsy-proven LN. The results showed that 3-pos patients had higher disease activity, renal histopathology indicative of more active disease, and a more rapid decline in the eGFR than those without 3-pos.

Disease activity, as measured by the SLEDAI-2K, was also greater in the 3-pos than non-3-pos patients. The high renal SLEDAI-2K score, but not the non-renal SLEDAI-2K score, in 3-pos patients suggests that renal disease activity is probably the main driver for their high global disease activity. Previous studies showed that anti-dsDNA, as a component of SLEDAI-2K, correlates with SLE disease activity over time and that rising titers are predictive of flares [16,17]. Similarly, anti-nucleosome titers were shown to correlate significantly with disease activity, as measured by validated global scoring systems such as the SLEDAI-2K [18]. Although still controversial, several lines of evidence support a significant association between anti-histone antibody titers and indices of disease activity [19,20]. Since the nucleosome is able to stimulate the formation of autoantibodies directed against all (anti-nucleosome) or parts (anti-dsDNA and anti-histone) of its structure, and these autoantibodies are frequently found simultaneously in lupus patients, co-positivity for anti-dsDNA, -nucleosome, and/or -histone is likely to be more strongly associated with lupus activity than is positivity for any one of these antibodies alone. In our study, 3-pos patients had higher SLEDAI-2K scores and lower C3 levels than non-3-pos patients, suggesting that the coexistence of these autoantibodies can serve as a marker of lupus disease activity, regardless of renal involvement, in addition to guiding the follow-up care of these patients.

Our results also showed that, with increasing antibody co-positivity, both the degree of histopathologically determined renal activity and frequency of proliferative LN according to the ISN/RPS classification significantly increased. Several studies have shown that individual autoantibodies, including anti-dsDNA, -nucleosome and/or -histone are strongly associated with LN activity and the risk of developing proliferative glomerulonephritis [7,21]. Because LN is a multi-autoantibody autoimmune condition [22], co-positivity for these nephritogenic autoantibodies is likely to reflect more active renal pathology than is positivity for only one antibody. In retrospective studies conducted by a single center in China, 3-pos in LN patients correlated significantly with the activity index but not with the chronicity index, as determined by renal biopsies, and with an increased frequency of proliferative LN according to the ISN/RPS classification [23,24]. However, those studies investigated the simple correlation coefficient between 3-pos reactivity and renal histopathologic findings; they did not assess the level of co-positivity according to histopathological severity. In our study, analysis of the level of co-positivity according to each component of the activity and chronicity indexes showed that the activity index, but not the chronicity index score, increased with the level of co-positivity. Thus, a higher proportion of patients with proliferative LN was seen when the level of co-positivity was higher. These findings suggest that, histopathologically, LN patients with 3-pos have more active renal disease those with positivity to none, one, or two of the target antibodies. Thus, an assessment of the co-positivity status in lupus patients will provide valuable insight into renal involvement.

In this study, after a median follow-up of 83.2 months, the eGFR of LN patients with 3-pos indicated a more rapid decline than in non-3-pos patients. A Chinese study from Peking University First Hospital reported that LN patients with co-positivity for anti-dsDNA and anti-C1q had a worse renal outcome than patients with single positivity [25]. Another Chinese study from the 1st and 2nd affiliated hospitals of Harbin Medical University reported worse renal outcomes in 3-pos than non-3-pos LN patients, as defined by a doubling of the serum creatinine level for at least 6 months [23,24]. In agreement with those studies, we found that, compared to non-3-pos patients, 3-pos patients had a more rapid decline of renal function as a result of strong renal disease activity. Those findings, together with our own, suggest that co-positivity for nephritogenic autoantibodies can serve as a predictor of a poor renal prognosis in LN patients. However, because the findings were obtained in Asian patients, they remain to be confirmed in LN patients of different races and ethnicities, and in those from other geographic regions.

In this study, the 3-pos group had lower lymphocyte counts and C3 levels compared to the non-3-pos group. As 3-pos patients had higher disease activity and more active renal histopathology, and absolute lymphocyte counts and complement levels were used as markers of disease activity, it is logical to assume that lymphopenia and hypocomplementemia would be more frequent in 3-pos patients. Unexpectedly, however, more patients were positive for anti-RNP in the 3-pos than the non-3-pos group. In earlier studies, anti-RNP was less frequent in LN patients compared to patients without LN, suggesting a protective role of LN [26,27]. Other studies have demonstrated that the presence of anti-RNP was not different between patients with and without LN [28,29]. In a British multivariate analysis, during a 12-year follow-up, anti-RNP was not associated with LN development, although there was a significantly higher proportion of anti-RNP positive patients in the LN group [30]. In contrast, in the LUMINA study group, anti-RNP was a significant predictor of LN occurrence among Hispanic and African-American SLE patients [31], consistent with our findings. Due to the contradictory results among studies, further studies are required to clarify the association between anti-RNP and LN.

Our study had several limitations. First, cross-reactivity may occur among anti-dsDNA, -nucleosome, and -histone antibodies because a nucleosome consists of a DNA segment wound around eight histone proteins. In this study, anti-nucleosome and anti-histone were separately measured using ELISA with the substrate of the human nucleosome and whole histone proteins (H1, H2A, H2B, H3, and H4). The sensitivity and specificity have been reported as 89.7% and 88.4% for anti-nucleosome, and 28.2% and 97.7% for anti-histone, respectively [32]. Autoantigens recognized by anti-nucleosome are mainly conformational neoepitopes derived from apoptotic nucleosomes; therefore, most anti-nucleosomes do not react with DNA or histones alone [33]. Similarly, because epigenetic modifications of exposed histones occurring through the formation of apoptotic blebs or neutrophil extracellular traps elicit an immune response [34], it is unlikely that anti-histone cross-react with dsDNA or nucleosome. For the anti-dsDNA, radioimmunoassay (RIA) was performed with an antigen of plasmid DNA. The sensitivity and specificity of anti-dsDNA were reported as 38.0% and 95.0%, respectively [35]. Since a different technique was used to detect anti-dsDNA, and because the RIA anti-dsDNA detects antibodies with high avidity to DNA, cross-reactivity among these antibodies was less likely to develop. However, anti-dsDNA was responsible for anti-nucleosome activity in 30% of the cases according to absorption assay, and similar associations may be found between anti-nucleosome and anti-histone [36]. In addition, because target antigens and the origins of their autoantibodies have not been clearly defined, it is difficult to entirely discount the possibility of cross-reactivity between these autoantibodies. Second, various factors, including intercurrent infection, dehydration, and drug use (e.g. non-steroidal anti-inflammatory drugs or antibiotics), can temporarily lead to renal function decline. Although we tried to calculate the sustained decline of renal function on an annual basis, we could not exclude the possibility of a temporary increase in serum creatinine levels or damage caused by intercurrent infection, dehydration, or drug use. Third, 3-pos patients had a more rapid decline of renal function than non-3-pos patients. However, this did not translate into changes of 1-year CR, relapse, or ESRD. Future studies should enroll patients of different races and ethnicities to explain the discrepancies between soft and hard outcomes. Fourth, we evaluated the anti-nucleosome and anti-histone autoantibodies using commercially available ELISA kits. Because validation tests of these autoantibodies were not performed, validation of these commercially available ELISA kits used in the present study may have increased the reliability of our results.

In summary, our study examined the clinical, histopathologic, and prognostic significance of 3-pos in patients with biopsy-proven LN. Our results showed that 3-pos patients had higher lupus disease activity, more histopathologically active renal disease, and a more rapid eGFR decline than in non-3-pos patients. Moreover, we showed that 3-pos co-positivity can be used not only as a marker of lupus and renal disease activity but also as a predictor of a poor renal outcome in LN patients.

## Supplementary Material

Supplemental Material

## Data Availability

The full original protocol and dataset can be accessed upon request for academic researchers by contacting Professor Shin-Seok Lee (shinseok@chonnam.ac.kr).
