# Supplementary material for: Significance of co-positivity for anti-dsDNA, -nucleosome, and -histone antibodies in patients with lupus nephritis
Source: Ann Med. 2023 Mar 10;55(1):1009–17. doi: 10.1080/07853890.2023.2187076 (PMC10795605; doi:10.1080/07853890.2023.2187076)
Supplement: Supplemental Material [file IANN_A_2187076_SM6241.docx]

**Supplementary Table.** Associations of autoantibodies with renal biopsy findings in patients with lupus nephritis.

|  | Anti-dsDNA | | | Anti-nucleosome | | | Anti-histone | | | Co-positivity (n) | | | | |
| --- | --- | --- | --- | --- | --- | --- | --- | --- | --- | --- | --- | --- | --- | --- |
|  | Positive  (n=86) | Negative  (n=16) | P value | Positive  (n=69) | Negative  (n=33) | P value | Positive  (n=54) | Negative  (n=48) | P value | 0  N=8 | 1  N=23 | 2  N=27 | 3  N=44 | P for trend |
| **Activity index** |  |  |  |  |  |  |  |  |  |  |  |  |  |  |
| Endo-capillary hypercellularity | 1.37±1.20 | 0.75±1.12 | 0.048 | 1.46±1.18 | 0.88±1.16 | 0.016 | 1.52±1.10 | 1.00±1.27 | 0.016 | 0.38±1.06 | 0.91±1.20 | 1.41±1.21 | 1.55±1.13 | 0.016 |
| Leukocyte infiltration | 0.65±0.73 | 0.31±0.48 | 0.092 | 0.65±0.74 | 0.48±0.62 | 0.325 | 0.78±0.77 | 0.40±0.57 | 0.008 | 0.13±0.35 | 0.48±0.59 | 0.56±0.64 | 0.77±0.80 | 0.086 |
| Sub-endothelial hyaline deposits | 1.02±1.07 | 0.63±0.88 | 0.089 | 1.01±1.14 | 0.85±1.03 | 0.185 | 1.28±1.02 | 0.77±1.03 | 0.009 | 0.38±1.06 | 0.91±0.99 | 0.89±0.97 | 1.32±1.07 | 0.045 |
| Fibrinoid necrosis/  Karyorrhexis | 0.92±1.08 | 0.38±0.81 | 0.035 | 1.01±1.14 | 0.45±0.75 | 0.011 | 1.19±1.18 | 0.44±0.74 | <0.001 | 0.0±0.0 | 0.43±0.73 | 0.81±0.92 | 1.20±1.23 | 0.002 |
| Cellular crescents | 0.48±0.94 | 0.44±0.81 | 0.893 | 0.54±1.01 | 0.33±0.69 | 0.328 | 0.52±0.79 | 0.42±1.05 | 0.190 | 0.0±0.0 | 0.30±0.70 | 0.89±1.31 | 0.39±0.72 | 0.022 |
| Interstitial inflammation | 0.87±0.43 | 1.13±0.61 | 0.060 | 0.88±0.47 | 0.97±0.47 | 0.391 | 0.93±0.51 | 0.90±0.42 | 0.779 | 1.00±0.53 | 0.87±0.46 | 1.07±0.47 | 0.82±0.45 | 0.152 |
| **Chronicity index** |  |  |  |  |  |  |  |  |  |  |  |  |  |  |
| Glomerular sclerosis | 0.38±0.57 | 0.81±0.75 | 0.016 | 0.41±0.55 | 0.55±0.75 | 0.504 | 0.46±0.66 | 0.44±0.58 | 0.997 | 0.75±0.70 | 0.35±0.57 | 0.67±0.78 | 0.32±0.47 | 0.092 |
| Tubular atrophy | 0.63±0.59 | 0.81±0.65 | 0.289 | 0.68±0.60 | 0.61±0.61 | 0.546 | 0.70±0.60 | 0.69±0.55 | 0.386 | 0.88±0.64 | 0.48±0.59 | 0.70±0.61 | 0.68±0.60 | 0329 |
| Interstitial fibrosis | 0.67±0.51 | 0.81±0.54 | 0.357 | 0.67±0.50 | 0.76±0.56 | 0.471 | 0.70±0.50 | 0.69±0.55 | 0.814 | 1.00±0.53 | 0.61±0.58 | 0.70±0.46 | 0.68±0.52 | 0.360 |
| Fibrous crescents | 0.08±0.27 | 0.06±0.25 | 0.797 | 0.09±0.28 | 0.06±0.24 | 0.645 | 0.07±0.26 | 0.08±0.28 | 0.863 | 0.13±0.35 | 0.04±0.21 | 0.07±0.27 | 0.09±0.29 | 0.868 |

Unless otherwise indicated, the values are mean ± standard deviation.
